# Performance of Fly Ash Geopolymer Concrete Incorporating Bamboo Ash at Elevated Temperature

**DOI:** 10.3390/ma12203404

**Published:** 2019-10-17

**Authors:** Shafiq Ishak, Han-Seung Lee, Jitendra Kumar Singh, Mohd Azreen Mohd Ariffin, Nor Hasanah Abdul Shukor Lim, Hyun-Min Yang

**Affiliations:** 1 Department of Architectural Engineering, Hanyang University, 1271 Sa 3-dong, Sangnok-gu, Ansan 15588, Korea; shafiq94@hanyang.ac.kr (S.I.); ercleehs@hanyang.ac.kr (H.-S.L.); 2 Innovative Durable Building and Infrastructure Research Center, Department of Architectural Engineering, Hanyang University, 1271 Sa 3-dong, Sangnok-gu, Ansan 15588, Korea; yhm04@hanyang.ac.kr; 3 Forensic of Engineering Centre, School of Civil Engineering, Universiti Teknologi Malaysia, Johor Bahru 81310, Johor, Malaysia; mohdazreen@utm.my; 4 Department of Structure and Materials, School of Civil Engineering, Universiti Teknologi Malaysia, Johor Bahru 81310, Johor, Malaysia; norhasanah@utm.my

**Keywords:** fly ash, bamboo ash, supplementary materials, geopolymer concrete, elevated temperature

## Abstract

This paper presents the experimental results on the behavior of fly ash geopolymer concrete incorporating bamboo ash on the desired temperature (200 °C to 800 °C). Different amounts of bamboo ash were investigated and fly ash geopolymer concrete was considered as the control sample. The geopolymer was synthesized with sodium hydroxide and sodium silicate solutions. Ultrasonic pulse velocity, weight loss, and residual compressive strength were determined, and all samples were tested with two different cooling approaches i.e., an air-cooling (AC) and water-cooling (WC) regime. Results from these tests show that with the addition of 5% bamboo ash in fly ash, geopolymer exhibited a 5 MPa (53%) and 5.65 MPa (66%) improvement in residual strength, as well as 940 m/s (76%) and 727 m/s (53%) greater ultrasonic pulse velocity in AC and WC, respectively, at 800 °C when compared with control samples. Thus, bamboo ash can be one of the alternatives to geopolymer concrete when it faces exposure to high temperatures.

## 1. Introduction

Production of Portland cement consumes energy and releases a massive volume of carbon dioxide (CO_2_) into the atmosphere but is still considered as a conventional binder owing to its excellent performance in most civil engineering applications [1]. In addition, in some instances, the production of the concrete with Portland cement is less durable in an aggressive environment and at high temperature conditions [2]. However, it was observed that the geopolymer has become a problem solver to all these issues [3]. High demand for conventional concrete, which is known as environmentally friendly concrete, can also solve landfill problems by leading to recycling and reusing waste material. These problems can be eliminated by utilizing the industrial waste products in construction purposes. Using waste products as a cementitious material in geopolymer concrete would also maximize its recycling potential throughout the industrial sector.

Geopolymer is an inorganic composite which is produced by synthesizing pozzolanic materials under highly alkaline hydroxide and/or alkaline silicate [4]. Apart from that, the geopolymer concrete has superb resistance to chemical attack and exhibits great ability against aggressive environments with a high amount of CO_2_, high content of sulfate, and acid resistance [5]. A previous study by Wallah and Rangan [6] concluded that geopolymer concrete revealed small changes in the length and few increases in mass after one-year of exposure to sulfate solution. Bakharev [7] has studied the properties of concrete in different concentrations of sulfate solution with different type of activators and found that the properties of the concrete depended on the quality of materials and activators. 

Geopolymer concrete can be used in various kinds of applications including as a fire resistant, sealants, concretes, ceramics, etc. It was reported that geopolymer concrete can withstand high temperature exposure [8,9]. Therefore, geopolymer concrete may possess a superior fire resistance compared to conventional concrete i.e., Ordinary Portland Cement (OPC).

Interest in using fly ash as a sustainable material in geopolymer concrete has increased since 2000 [10]. Hardjito and Rangan [11] have investigated the effects of alkaline parameters, water content, and curing conditions in their research. In Malaysia, some researchers focused on geopolymer concrete as well [12,13]. As a result, geopolymers have become prominent among researchers due to its environmentally friendly and high performance characteristics.

Agriculture waste is a serious environmental problem in many countries. This waste is being mainly produced from gardens and rice fields. The majority of previous research involving agriculture waste involved Palm Oil Fuel Ash (POFA) [14,15,16] and Rice Husk Ash (RHA) [17,18,19] as binders. Navid Ranjbar et al., [20] have conducted an experiment regarding the performance of POFA and fly ash (FA) based geopolymer mortar exposed to elevated temperatures. It was concluded by them that all FA/POFA based geopolymers gained strength when exposed to temperatures up to 500 °C. However, by increasing FA content in samples, they produced higher compressive strength at 300 °C, while on the other hand increasing POFA content delays attaining maximum strength. They have also suggested that when the temperature was increased above 500 °C, all samples lost their strength. Besides this, a study was conducted which focused on the effect of pretreatment of FA and POFA on mechanical properties after the geopolymerization process [21]. It was shown that when FA is heated up to 800 °C, sintering of the particles was observed which led to a deformation and reactivation, thus leading to a reduction in the setting time and increased early compressive strength.

Interest in bamboo for construction has grown continuously as the focus shifts towards reducing the environment impact and embodied energy of the built environment. For developing countries, bamboo is considered as an ideal crop for rural development. Bamboo production and utilization are considered relevant to many in the UN for sustainable development goals. Naturally, the bamboo is found in cylindrical pole or culm. Bamboo is also part of the grass family. There are over 1200 species of bamboo all over the world, with structural species varying by locations. The different species can be categorized into three types of root systems: sympodial (clumping), monopodial (running), and amphipodial (clumping and running). According to the Food and Agriculture Organization of the United Nations (UNFAO), a total of 72% of land area in Malaysia is filled with forests. Bamboo is an easy plant to grow. Tropical rainforest areas found in Malaysia provide ideal growing conditions for the bamboo plant. The production of bamboo charcoal has increased and its applications especially in healthcare, cooking, water purification, and gardening have grown significantly [22]. Consequently, bamboo ash is the waste from the production of bamboo charcoal. Although rich in silica, the poor performance of bamboo ash is owing to the presence of silicate material. Due to the absence of alumina, it is attractive in combination with other materials which are rich in alumina i.e., fly ash. Commonly found in Malaysia and Indonesia, bamboo plant has been used as a fire protection material [23,24]. Bamboo is fire resistant even at higher temperatures, thus, it can be used in the construction industry.

There is lack of research on the use of bamboo ash in the construction industry. Therefore, in present study, we have experimentally investigated the properties and performance of fly ash geopolymer concrete incorporating with bamboo ash under elevated temperature. In addition, there is no previous study that has used bamboo ash as a construction material. This investigation includes the effect of physical appearances, compressive strength, weight loss, and ultrasonic pulse velocity loss after the desired exposure.

## 2. Experimentation

### 2.1. Materials

#### 2.1.1. Binder

In this study, fly ash (FA) as low calcium fly ash (class F) and bamboo ash was used. The chemical composition and particle size analysis for both materials will be discussed in the results and discussion section.

Fly ash was obtained from Tanjung Bin, Johor, Malaysia. The bamboo ash (BA) was obtained from Lanchang, Pahang, Malaysia. First, collected bamboo ash was dried in the oven for 24 hours at 110 °C (± 5 °C) to ensure that there was no moisture available. Then, the bamboo ash was ground in an abrasion test machine for 6 hours to improve the fineness of the ash. Then, the bamboo ash was sieved through a 45 μm sieve in order to remove bigger size of ash particle and impurities. Only the fine bamboo ash passing through the sieve were collected following the standard size of Portland cement used in the mixing. The specific gravity of FA and BA was 2.20 and 2.05, respectively.

#### 2.1.2. Aggregates

The standards used to determine the properties of aggregates are ASTM C127 [25], ASTM C128-15 [26], ASTM C29 [27], and BS EN 933-1:2012 [28]. ASTM C127 was used to determine the specific gravity and water absorption of coarse aggregate. ASTM C128 was used to obtain specific gravity and water absorption of fine aggregate. Apart from that, ASTM C29 was conducted to acquire the bulk density of both aggregates and BS EN 933-1:2012 [28] was used to check the grading requirement of the aggregates.

In this research, crushed granite with nominal size of 10 mm was used as a coarse aggregate. The specific gravity, water absorption, and bulk density of the coarse aggregate was 2.7, 0.5%, and 1551 kg/m^3^, respectively. River sand was used in fine aggregates and obtained from a local source in Johor, Malaysia. Specific gravity, water absorption, and bulk density of the fine aggregate was 2.6, 0.7%, and 1650 kg/m^3^, respectively. To ensure the aggregates did not absorb alkaline solution during the mixing process, saturated surface dry (SSD) conditions for both aggregates were conducted. For this purpose, both coarse and fine aggregates were soaked separately with clean tap water. Then, the aggregates were placed on a plastic sheet until the surface became dry.

#### 2.1.3. Alkaline Solution

Alkali Sodium based activator purchased from QReC^TM^, Auckland, New Zealand was used in this research. The alkaline solution was prepared by mixing 10 M sodium hydroxide (NaOH) with sodium silicate (Na_2_SiO_3_). The activator to binder ratio was different starting from 0.40, 0.45, and 0.5. Optimization of the activator to binder ratio was carried on the basis of workability results. 

#### 2.1.4. Superplasticizer

In this research, Master Glenium ACE 8589 (Master® Builders Solutions, Kuala Lumpur, Malaysia) was used as a superplasticizer (SP). SP is a chemical admixture that was added to the concrete during the mixing process. It is also known as a water reducer. SP provides exceptionally good early strength development and maintains flowability for a considerable period of time. Behzad and Jay have studied the effect of different SPs on the workability and strength of fly ash based geopolymer and they have found that SP is effective in improving the properties of concrete, which are directly dependant on the type of activator and the SP [29].

### 2.2. Mix Proportions

The preliminary mix design was optimized based on several factors. In this study, the mass ratio of binder to aggregates and coarse to fine aggregates was set to be 1. The mass ratio for coarse to fine aggregates and sodium silicate to sodium hydroxide was set to be 1 and 2.5, respectively, along with 3% superplasticizer. Huseien et al. [30], have found that this ratio for binder to aggregates provided optimum results of flowability, compressive strength, and bending stress. Besides, increased binder content led to reducing the workability, strength of samples, and reduced initial and final setting times of the geopolymer.

Table 1 shows the mix proportion used in this research. The mixing phase was an important part in the production of geopolymer concrete. The mixing process and curing was carried out at 25 ± 2 °C. This proportion is generally being used for ultra-high performance fiber reinforced concrete [31,32,33,34,35]. Huseien et al. have considered 1100 kg/m^3^ binder content in alkali activated mortar for durability properties [36] and they found that this composition has increased the durability performance of mortar. In present study, this proposed mix proportion was not acceptable, but we have tried to consume the maximum waste utilized in the construction industry. Therefore, we have followed the Huseien et al. mortar proportion for the present study [36]. During mixing of the proposed proportion, it was very hard to mix the geopolymer concrete properly however, when using 3% SP it was easy to mix. 

### 2.3. Flow Table Test

The determination for the workability of the geopolymer mortar was conducted in accordance with ASTM C1437 [37]. The standard conical frustum with a 100 mm diameter was used. Table 2 shows the workability criteria.

### 2.4. Compressive Strength Test

The compressive strength test measurement was carried out on a 100cm × 100cm × 100cm concrete sample. This test was conducted according to ASTM C109-16 standard [38].

### 2.5. Testing Procedures

#### 2.5.1. X-ray Fluorescence (XRF)

The chemical compositions of bamboo ash and fly ash were acquired by XRF test (Rigaku NEX CG, Tokyo, Japan). 100 grams of both fly ash and bamboo ash was sealed safely in a plastic bag before being tested.

#### 2.5.2. X-ray Diffraction (XRD)

X-ray Diffraction (XRD: Rigaku Smartlab Diffractometer) of fly ash, bamboo ash, 100% fly ash and 5% bamboo ash + 95% fly ash geopolymer concrete after 7 days of curing were carried out from 2θ = 5–85° using Cu radiation (λ = 1.54182 Å) at 25 kV.

#### 2.5.3. Particle Size Analysis (PSA)

Particle size analysis (PSA) was conducted to investigate the size distribution of the binder. This analysis was performed by laser scattering technique (Mastersizer 3000). This test was carried out using the wetting method where the particles were dispersed using distilled water to avoid an agglomerated condition.

#### 2.5.4. Scanning Electron Microscopy (SEM)

The surface morphology of both materials (bamboo ash and fly ash) as well as 100% fly ash and 5% bamboo ash+95% fly ash geopolymer concrete after 7 days of curing were conducted by scanning electron microscopy (SEM, Jeol, Tokyo, Japan) operated at 15 kV.

#### 2.5.5. Ultrasonic Pulse Velocity (UPV) Test

The UPV value of concrete samples was determined according to BS EN 12504-4:2004 [39] after exposure to high temperature.

#### 2.5.6. Fire Endurance Test

**Figure 1:**
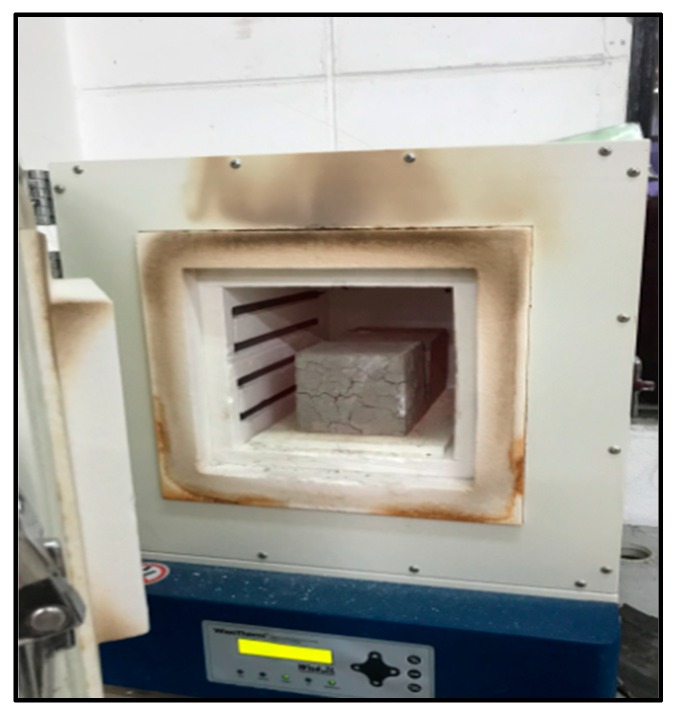
Overview of an automatic electrical furnace.

The fire endurance test was carried out in accordance with ASTM E119-12a [40] using automatic electrical furnace (Figure 1) with 100 mm × 100 mm × 100 mm concrete. All samples were cured for 28 days at 25 ± 2 °C before being subjected to high temperature. An electrically-heated furnace designed for a maximum 1200 °C was used. All samples were heated for the duration of 1 hour at 200 °C, 400 °C, 600 °C and 800 °C as targeted temperature. Two different cooling approaches were tested which were air cooling (AC) and water cooling (WC) regimes with curing conditions at 25 °C and 60% relative humidity. Different cooling regimes for normal cement composite had a significant influence on the mechanical properties of the concrete after exposure [41,42]. Before the test were conducted, all samples were weighed to determine their initial density and initial ultrasonic pulse velocity (UPV) value. After acquiring the temperature level, a further experiment was then carried out to determine the UPV loss, weight loss, physical appearances, and residual compressive strength. 

## 3. Results and Discussion

### 3.1. Characterization of the Binder

#### 3.1.1. X-ray Fluorescence (XRF)

The chemical composition of bamboo ash and fly ash were determined using XRF. Based on the results shown in Table 3, the main oxides composition of bamboo ash is silica, potassium oxide, calcium oxide, and sulfur trioxide containing 35.2%, 33.1%, 13.5%, and 8.3%, respectively. For fly ash, most of the compounds are silica and alumina. Furthermore, silica/alumina ratio for bamboo ash is unidentified while fly ash is around 2.0. Silica and alumina are very important for geopolymer synthesis. A nil percentage of alumina in bamboo ash is too unrealistic for geopolymerization to occur. The presence of high amount of calcium oxide in bamboo ash reduces the setting time of fly ash containing geopolymer concrete. The fly ash used in the present study was considered as Class F, revealing that the summation of silica, alumina and iron oxide is more than 88%.

#### 3.1.2. X-ray Diffraction (XRD)

**Figure 2:**
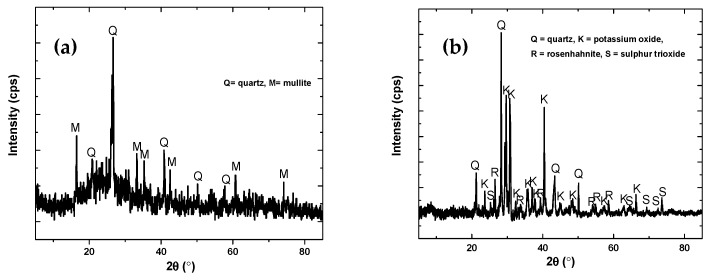
X-ray Diffraction (XRD) of (**a**) fly ash and (**b**) bamboo ash.

XRD of both binder materials is shown in Figure 2. Fly ash shows the presence of quartz (SiO_2_, JCPDS = 88-2487) and mullite (3Al_2_O_3_.2SiO_2_, JCPDS = 06-0259) in the XRD pattern (Figure 2a), while bamboo ash diffraction patterns show the presence of quartz (JCPDS = 88-2487), potassium oxide (K_2_O, JCPDS = 26-1327), rosenhahnite i.e., calcium hydroxide silicate (Ca_3_Si_3_O_8_(OH)_2_, JCPDS = 83-1242), and sulfur trioxide (SO_3_, JCPDS = 76-0760) (Figure 2b). Potassium oxide, rosenhahnite, and sulfur trioxide are found only in bamboo ash. This result corroborates with XRF data (Table 3). A sharp peak at 28.28° indicates a mainly crystalline structure consisting of quartz, as well as potassium oxide and rosenhahnite in bamboo ash. 

#### 3.1.3. Particle Size Analysis

**Figure 3:**
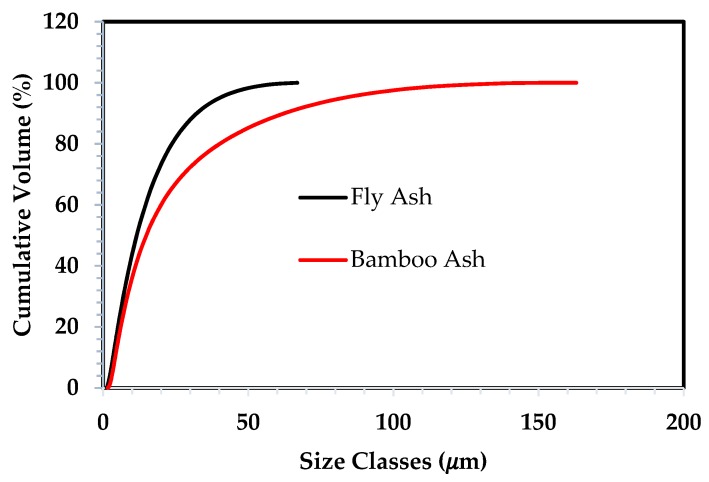
Particle size analysis of fly ash and bamboo ash.

The particle size distribution of fly ash and bamboo ash was characterized by their D50 values as shown in Figure 3. The particle of bamboo ash is in micro size where the biggest size is determined at 163 μm with a median size of 14.5 μm. Also, the biggest size of particles in fly ash is revealed to be 66.9 μm with a median size of 15 μm. It is revealed that 83% of the bamboo ash particles are smaller than 45 μm, while almost 96% of fly ash particles have a size less than 45 μm. The specific area is found to be 510.4 and 658.4 m^2^/kg for bamboo ash and fly ash particles, respectively.

#### 3.1.4. Scanning Electron Microscopy (SEM) Analysis

**Figure 4:**
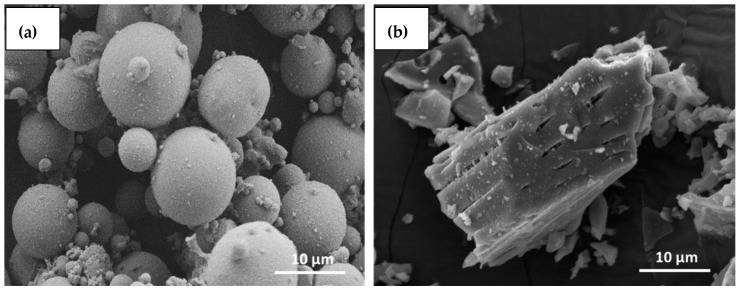
Scanning Electron Microscopy (SEM) micrographs of (**a**) Fly ash (**b**) Bamboo ash.

Figure 4 shows the surface and shape morphology of the fly ash as well as the bamboo ash used in this study. Most of the fly ash particles have a glassy and spherical structure (Figure 4a), and are also known as cenospheres [43]. This ash contains a series of spherical vitreous particles of different sizes. Meanwhile, Figure 4b shows the SEM micrographs of bamboo ash, which depicted that bamboo ash has a rectangular structure and different particle sizes. It also has a porous structure, as can be seen from Figure 4b.

### 3.2. Appropriate Mix Proportion

#### 3.2.1. Workability

Table 4 represents the workability result of geopolymer concrete using different mix proportions. The standard deviation of the different mix proportions is found to be 2–8 mm. It is depicted that the slump value increases with the increase of the activator to binder ratio. The mix was very stiff and no flow was observed when the activator to binder ratio was 0.40 for both types of sample. Based on the visual observation, the mix was not homogenous and difficult to mix and compact. Apparently, the bamboo ash absorbs more water during the mixing, thus reducing the workability of the mix.

Once the activator to binder ratio was reached at 0.45, the slump value increases compared to 0.40 for 100% FA (Csample) and 5% BA+ 95% FA (Asample). Based on the observations, the mix was quite homogenous and the mixing process was easier compared to for the 0.40 ratio. The workable mix with the highest flow value was obtained when the activator to binder ratio was 0.50 for both samples. 

In conclusion, the small percentage i.e., 5% addition of bamboo ash at a 0.45 ratio, gave a much smaller slump loss in Asample compared to Csample. However, with 0.40 and 0.50 ratios, the slump loss is 10 mm for Asample. Thus, we decided to perform another experiment in a 0.45 activator to binder ratio. 

#### 3.2.2. Compressive Strength

Table 5 shows the compressive strength as well as the standard deviation of samples with curing duration. This table shows the effect of bamboo ash to fly ash ratio (%) on the compressive strength of geopolymer concrete at a 0.45 activator to binder ratio. The results show that the compressive strength of geopolymer concrete is increased with the increasing of curing time. This is due to the geopolymerization process between alumina and silica from the binder with the alkaline solution. But our studies failed to get desired ultra-high performance fiber reinforced concrete compressive strength [31,32,33,34,35]. However, as the percentage of bamboo ash increased, the compressive strength of the geopolymer concrete was decreased. This was probably due to a lack of the amount of alumina in bamboo ash, which reduced the geopolymerization product and contributed to the strength of concrete [44]. For 7 days of curing, replacement with 5% bamboo ash shows higher compressive strength at 18.94 MPa compared to 100% of fly ash i.e., 16.15 MPa. The increased early age compressive strength of BA with FA is due to the hydrolysis which imposed to form crystalline phases and reduced the porosity of the samples. Besides, it was also concluded that surface area of the mixture was increased due to the addition of bamboo ash, since the specific gravity of BA (2.05) is less than FA (2.20).

The standard deviation in the compressive strength for different mix proportions was found to be 0.11 to 1.31 MPa. A very stable proportion was needed in order to obtain a very good concrete mix for construction purposes. The addition of 5% bamboo ash was shown to be the most suitable mixture to get a better performance in regards to early compressive strength compared to other mixtures.

**Figure 5:**
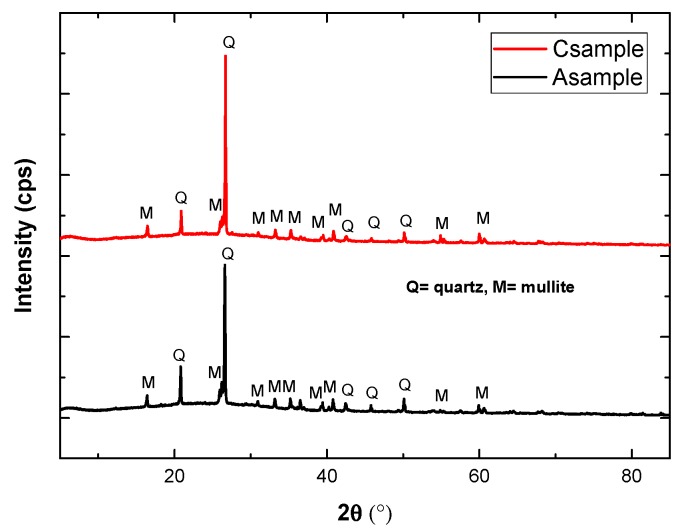
XRD analysis for Csample (100% FA) and Asample (5% BA + 95% FA) after 7 days of curing.

**Figure 6:**
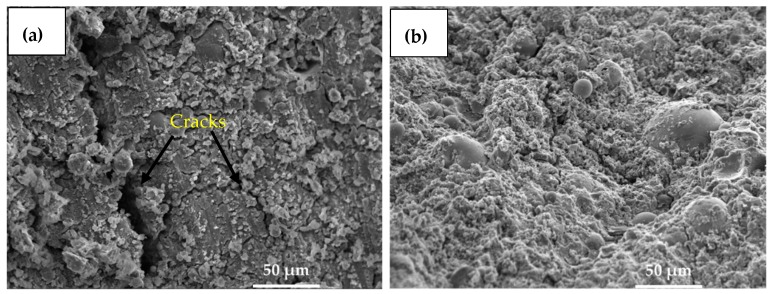
SEM micrographs of (**a**) 100% fly ash (Csample) and (**b**) 5% bamboo ash + 95% fly ash (Asample) after 7 days of curing.

To understand the formation of phases after 7 days of curing, XRD of Csample (100% FA) and Asample (5% BA + 95% FA) was performed and results are shown in Figure 5. Quartz and mullite were obtained in both samples. There is a possibility that bamboo ash in Asample was completely mixed and dissolved in the matrix of fly ash attributed to the high dissolution rate of potassium oxide, rosenhahnite ,and sulfur trioxide in high alkaline condition, as explained by other researchers [45], during 7 days of curing. Thus, only quartz and mullite are found as present in 100% fly ash. This result confirms that owing to the dissolution of oxides present in bamboo ash control, the crack formed and filled out the porosity of geopolymer concrete (SEM results will be shown in Figure 6), leading to higher compressive strength being observed for 5%BA + 95%FA (Table 5) after 7 days of curing. The presence of quartz and mullite in Csample and Asample explained that SiO_2_ and Al_2_O_3_ are not fully utilized for geopolymer formation. Bamboo ash and activator are the materials that are involved in the synthesis of fly ash based geopolymer concrete. The pure geopolymer network actually consists mainly of Si, Al, and O with alkali Na^+^ or K^+^. In this reaction, all minerals did not participate in the geopolymerization process. 

Figure 6 shows the SEM results of 100% fly (Csample) and 5% bamboo ash + 95% fly ash (Asample) after 7 days of curing at 0.45 activator to binder ratio. 100% FA (Csample) containing sample shows macro and micro cracks in Figure 6a, however, 5% bamboo ash + 95% fly ash (Asample) does not show any defect in Figure 6b which indicated that the potassium oxide, sulfur trioxide, and rosenhahnite has dissolved and filled out the cracks/pores in the concrete. Thus, after 7 days, the 5% bamboo ash + 95% fly ash sample shows higher compressive strength (Table 5). The bamboo ash has the property to hold the paste together, thus controlling the cracks within the concrete owing to the reactive compositions which contain potassium oxide and other oxides. Generally, previous researchers [46] agreed with the addition of fiber into concrete, which increases the strength of the concrete structure. Moreover, Csample exhibits cracks, pores, and defects, which decreases the positive properties of concrete.

### 3.3. Fire Endurance Test

#### 3.3.1. Cooling Effect on the Physical Appearance of the Concrete 

High temperature i.e., heat exposure is one of the most important parameters which affects the surface characteristics, surface outlook, shape, and color of concrete. Although it does not give significant information regarding the distortion suffered by the concrete, it will give an immediate impression of the failure tendency of the concrete. Table 6 and Table 7 represent a detailed picture of the concrete after exposure to different temperature and cooling regimes for Csample and Asample, respectively.

It can be observed from the entire cooling regime at 200 °C that the color of the sample was grey for both Csample and Asample with a smooth and sharp edges. These characteristics are maintained up to 400 °C. At 600 °C, the light grey color is observed for air cooling (AC) with rough surface while water cooling (WC) exhibits yellowish grey with cracks appeared in Csample whereas in Asample there is no cracks. At 800 °C, the Csample develops heavy cracks throughout the surface in AC and WC, whereas Asample shows lesser cracks compared to Csample in both AC and WC. 

The change in the matrix structure is a consequence of the dehydration in the phases of the binder during exposure to different temperatures. It is manifested as changes in porosity and color, as well as physical defects such as the presence of cracks. It is matched with the results presented in Table 6 and Table 7 for Csample and Asample, respectively, which show the physical appearance of the concrete after exposure to high temperature. In general, the color changes experienced in both samples as a result of heating may be linked to the chemical transformations taking place in the heated samples. 

#### 3.3.2. Residual Compressive Strength

The residual compressive strength of Csample and Asample at different temperature and cooling regimes is shown in Table 8. For both cooling types, the residual compressive strength increases at 200 °C and achieved a maximum strength for Csample and Asample. At 400 °C, the Csample has the least strength loss of 41% and 43% for AC and WC, respectively. For 600 °C, the loss trend continues to increase for both types of samples and achieved a maximum loss of compressive strength at 800 °C. 78% (AC) and 80% (WC) of strength loss gained by Csample which is the highest loss of compressive strength, compared with Asample with 65% (A/C) and 66% (W/C) loss.

According to a previous researcher [40], geopolymer concrete strength was enhanced in heat conditions. Changes in chemical structure and the dehydration of free and chemically-bound water was caused from the exposure to high temperature. As the temperature increases, the moisture particles inside the samples tend to escape to the surface. 

In this research, it was proved that fly ash geopolymer concrete (Asample and Csample) does not complete a geopolymerization process until 28 days have passed. Within 200 °C exposure, both type of samples gain an improvement in terms of strength and also the matrix structure. These are associated with the reported changes in the value of compressive strength after exposure. 

It is seen that the water molecule is expelled from the geopolymer concrete during the presence of heat, which improves the strength of the concrete and also leads to the discontinuous nano-pores of the matrix. It is possible that not all water molecules were expelled due to high temperature, especially in a larger sample. Higher surface tensions in larger samples will dissipate moisture at slower rates compared to in smaller samples. 

Starting from 400 °C, 600 °C and 800 °C levels of heat exposure, the the compressive strength decreased for both type of samples. But in the presence of bamboo ash, the residual compressive strength of Asample tends to show a positive result at 800 °C where the strength is higher compared to Csample. 

This clearly shows that at 800 °C, the presence of bamboo ash contains potassium oxide, thus producing a significant compressive strength compared to Csample. If alkali metal oxides content is above a certain limit, it exhibits a high coefficient of thermal expansion. With the presence of bamboo ash at high temperature, the tendency of the oxides lead to change its shape to have a higher area and volume. Thus, more solid and packed molecules are formed and provide better structural components for the geopolymer concrete. 

#### 3.3.3. Ultrasonic Pulse Velocity (UPV) Value after Exposure 

The change in UPV value due to exposure to high temperature is depicted in Table 9. The UPV values at the initial temperature (27 °C) were 3854 m/s (AC) and 3850 m/s (WC) for Csample, and 3810 m/s (AC) and 3802 m/s (WC) for Asample. UPV value increases at 200 °C yielded values of 4451 m/s (AC) and 4417 m/s (W/C) for Csample, and 4438 m/s (AC) and 4394 m/s (WC) for Asample, which is the highest value among all studied temperature_._ At 400 °C, the trends changed, after which the Csample experienced UPV losses of 13.3% (AC) and 14.0% (WC) compared to Asample, which experienced 12.9% (AC) and 16.1% (WC) UPV losses. At 600 °C, the loss trends continued to increase and achieved a maximum loss at 800 °C for both types of samples. 67.8% (AC) and 64.7% (WC) losses was achieved by Csample, while 42.8% (WC) and 45.1% (AC) were achieved for Asample_._ The Asample exhibited a smaller loss of UPV compared to Csample_._ Generally, the reduction of the velocity in the concrete was due to the deformation of the microstructure in the geopolymer concrete. A rise in the temperature increases the amount of air voids in the concrete samples. Thus, the transmission speed of sound waves decreased with the increase in the traveling time of the ultrasonic pulse transmission.

The quality of the concrete can be classified based on Table 10 [47]. Based on Table 10 values, good quality concrete can be produced at 200 °C. It was proven that the highest compressive strength corresponded with the highest UPV value. Apart from that, at 800 °C, Asample gained a higher UPV value compared to Csample, which corresponded with the residual compressive strength results.

#### 3.3.4. Weight Loss of Concrete 

The effect of elevated temperature on the weight loss of geopolymer concrete for all curing regimes is depicted in Table 11. The initial weight of the samples is expressed as the density of the sample exposed to a 25 °C temperature. The weight loss of Csample and Asample increased as the temperature increased. The weight loss increased significantly from 200 °C to 400 °C where the mass loss observed was a result of moisture movement out of the geopolymer matrix. The reduction in weight loss gradually decreased from 11.96% to 10.83% for AC to WC, respectively, in Csample at 600 °C. However, a greater loss is to be found at 800 °C with ranges of 12.3% (AC) and 10.79% (WC). Apart from that, the weight loss for Asample was slightly higher compared to Csample at 800 °C, having reached 12.62% (AC) and 11.28% (WC). The exposure to elevated temperature can lead to changes in the stiffness and mechanical properties of geopolymer concrete. It was proved that high temperature can affect the stiffness and mechanical properties of geopolymer concrete [48].

In addition, an increase in temperature and weight reduction lead to deterioration of the structural integrity of geopolymer concrete. Asample yielded a higher weight loss compared to Csample. Furthermore, weight loss was observed to be lower in WC compared to AC regime. This may be due to water being absorbed during the application of water-cooling to make sure the temperature of the concrete was under control.

**Figure 7:**
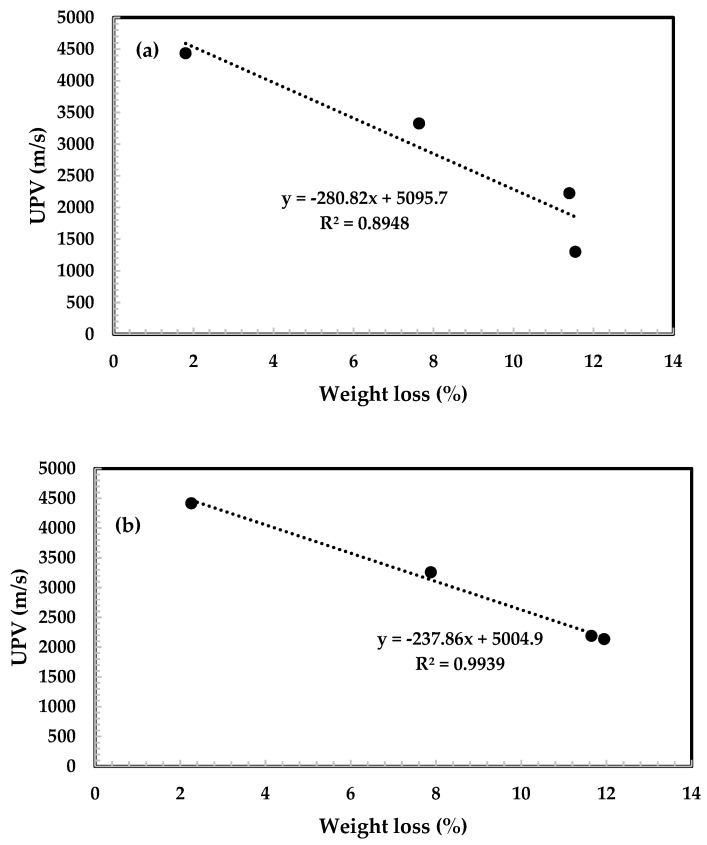
Ultrasonic pulse velocity (UPV) value of (**a**) Csample and (**b**) Asample as a function of weight loss.

The experimental results indicated that Csample had a lower confidence level i.e., R^2^ = 0.8949 (Figure 7a) that lay outside of the recommendation range (0.90–1.00) whereas Asample (Figure 7b) showed a strong relationship between these two parameters with a high confidence level i.e. 0.9939. From this result, it is illustrated that the experimental results show fitted well for Asample but not for Csample. Thus, it can be concluded that the presence of 5% bamboo ash in 95% fly ash gives a better confidence level compared to the control sample.

## 4. Conclusions

Based on the experimental results and discussion, the following conclusions can be drawn:

1. SEM analysis shows a rectangular shape of bamboo ash and it is believed that it can hold the paste together and help to provide resistance at high temperatures.
2. A 5% addition of bamboo ash in 95% fly ash provided better compressive strength after a short period of time i.e., 7 days of curing, compared to the control sample.
3. A light grey color was observed for air cooling (AC) at 600 °C with a rough surface and cracks that appeared in Csample during water cooling (WC), whereas there were no cracks in Asample.
4. Csample and Asample gained approximately 36% and 39% residual compressive strength at 200 °C of exposure, respectively, whereas losses of approximately 62% and 41% from 400 °C to 800 °C, respectively, were found after using the air cooling regime.
5. Asample exhibited the highest residual compressive strength at 800 °C of exposure compared to Csample for the AC and WC regimes.
6. UPV values for both types of samples increased tremendously at 200 °C of exposure, which was concluded to be good in terms of concrete quality. The UPV values tended to decrease from 400 °C to 800 °C of concrete exposure. A greater loss was found at 800 °C of exposure and it led to a change in the stiffness and mechanical properties of geopolymer concrete.
7. Asample shows a high confidence level and the best fitted value compared to Csample in UPV vs. weight loss results, which reveals that the velocity of heat passing through the concrete is slower.

## Figures and Tables

**Table 1:** Mixed proportion of fly ash geopolymer concrete incorporated with bamboo ash.

No.	Fly Ash (kg/m^3^)	Bamboo Ash (kg/m^3^)	NaOH (kg/m^3^)	Na_2_SiO_3_ (kg/m^3^)	Activator/Binder	Water (kg/m^3^)
1.	1215	–	138.86	347.14	0.40	63.18
2.	1154	61(5%)	138.60	347.14		63.18
3.	1093	122(10%)	138.60	347.14		63.18
4.	1033	182(15%)	138.60	347.14		63.18
5.	972	243(20%)	138.86	347.14		63.18
6.	729	486(40%)	138.86	347.14		63.18
7.	486	729(60%)	138.86	347.14		63.18
8.	243	972(80%)	138.86	347.14		63.18
9.	–	1215(100%)	138.86	347.14		63.18

**Table 2:** Workability criteria for geopolymer mortar.

No	Flow Diameter	Workability
1	Above 250 mm	Very High
2	180 mm to 250 mm	High
3	150 mm to 180 mm	Moderate
4	150 mm to 120 mm	Stiff
5	Below 120 mm	Very Stiff

**Table 3:** Chemical composition (%) of BA and FA.

Chemical Compounds	BA	FA
Silica (SiO_2_)	35.2	55.92
Alumina (Al_2_O_3_)	–	28.8
Calcium oxide (CaO)	13.5	5.16
Potassium oxide (K_2_O)	33.1	0.94
Sulfur trioxide (SO_3_)	8.3	–
Iron oxide (Fe_2_O_3_)	1.0	3.67
Manganese oxide (MnO)	3.1	–
Phosphorus pentaoxide (P_2_O_5_)	–	0.69
Titanium oxide (TiO_2_)	–	2.04
Magnesium oxide (MgO)	–	1.48
Loss of Ignition (LOI)	5.8	1.3

**Table 4:** Slump test result.

Sample	Activator to Binder Ratio	Slump Value (mm)			Average (mm)	Standard Deviation (mm)	Slump Loss (mm)
		Sample 1	Sample 2	Sample 3			
100% FA (Csample)	0.40	140	142	138	140	2.00	0
	0.45	202	187	190	193	7.94	0
	0.50	225	220	215	220	5.00	0
5% BA+ 95% FA (Asample)	0.40	139	126	125	130	7.81	10
	0.45	185	183	187	185	2.00	8
	0.50	219	203	208	210	8.19	10

**Table 5:** Effect of curing period on compressive strength of the bamboo ash (BA) to fly ash (FA) ratio (%) in the 0.45 activator to binder ratio.

Sample ID	Curing Time (Days)	Compressive Strength (MPa)			Average (MPa)	Standard Deviation (MPa)
		Sample 1	Sample 2	Sample 3		
100FA	7	16.31	16.19	15.96	16.15	0.18
	14	32.48	33.76	33.93	33.39	0.79
	28	42.81	42.53	41.06	42.13	0.94
5BA:95FA	7	18.67	19.1	19.05	18.94	0.24
	14	31.01	30.34	32.14	31.16	0.91
	28	42.56	41.03	39.96	41.18	1.31
10BA:90FA	7	15.87	16.03	16.21	16.04	0.17
	14	24.52	25.1	24.86	24.83	0.29
	28	39.44	40.12	39.68	39.75	0.34
15BA:85FA	7	13.1	14.05	13.85	13.67	0.50
	14	22.66	23.81	23.06	23.18	0.58
	28	32.28	33.14	33.56	32.99	0.65
20BA:80FA	7	11.01	12.56	12.14	11.90	0.80
	14	21.41	22.35	21.03	21.60	0.68
	28	34.8	35.24	34.78	34.94	0.26
40BA:60FA	7	7.5	6.96	8.5	7.65	0.78
	14	6.86	8.96	7.48	7.77	1.08
	28	9.21	10.04	9.86	9.70	0.44
60BA:40FA	7	2.35	2.37	2.5	2.41	0.08
	14	5.12	5.74	6.01	5.62	0.46
	28	7.03	7.56	8.05	7.55	0.51
80BA:20FA	7	0	0	0	0	0.00
	14	1.06	1.24	1.33	1.21	0.14
	28	2.44	2.22	2.29	2.32	0.11
100BA	7	0	0	0	0	0.00
	14	0	0	0	0	0.00
	28	0	0	0	0	0.00

**Table 6:** Cooling type, color, and texture of Csample.

Parameters	Temperature (°C)							
	200		400		600		800	
Type of cooling	AC	WC	AC	WC	AC	WC	AC	WC
Color	Grey	Grey	Grey	Grey	Light grey	Yellowish grey	Light grey	Grey
Texture	Smooth	Smooth	Smooth	Smooth	Rough	Crack	Fragmentation	Fragmentation

**Table 7:** Cooling type, appearance, color, and texture of Asample.

Parameters	Temperature (°C)							
	200		400		600		800	
Type of cooling	AC	WC	AC	WC	AC	WC	AC	WC
Color	Grey	Grey	Grey	Grey	Light grey	Light grey	Grey	Grey
Texture	Smooth	Smooth	Smooth	Smooth	Rough	Rough	Rough	Fragmentation

**Table 8:** Residual compressive strength of samples exposed at different temperature.

Temperature (°C)	Residual Compressive Strength (MPa)			
	Csample (100% FA)		Asample (95% FA + 5% BA)	
	AC	WC	AC	WC
27	42.6	42.8	41.1	41.5
200	57.93	56.10	57.28	55.82
400	25.14	24.43	24.69	22.48
600	15.40	14.70	15.12	14.89
800	9.47	8.54	14.51	14.19

**Table 9:** UPV value after exposure to different temperatures.

Temperature (°C)	UPV (m/s)			
	Csample		Asample	
	AC	WC	AC	WC
27	3854	3850	3810	3802
200	4451	4417	4438	4394
400	3340	3314	3320	3191
600	2241	2210	2235	2140
800	1240	1360	2180	2087

**Table 10:** Quality of concrete based on UPV.

UPV (m/s)	Quality of Concrete
>4500	Excellent
3500–4500	Good
3000–3500	Doubtful
2000–3000	Poor
<2000	Very Poor

**Table 11:** Weight loss of concrete samples.

Sample	Temperature (%)	Type of Cooling	Weight Loss (%)
Csample	200	AC	2.41
		WC	1.19
	400	AC	8.9
		WC	6.38
	600	AC	11.96
		WC	10.83
	800	AC	12.3
		WC	10.79
Asample	200	AC	2.89
		WC	1.62
	400	AC	9.1
		WC	6.65
	600	AC	12.09
		WC	11.21
	800	AC	12.62
		WC	11.28
